# On-line plasticity in spoken sentence comprehension: Adapting to time-compressed speech

**DOI:** 10.1016/j.neuroimage.2009.07.032

**Published:** 2010-01-01

**Authors:** Patti Adank, Joseph T. Devlin

**Affiliations:** a School of Psychological Sciences, University of Manchester, Manchester, UK; b Donders Institute for Brain, Cognition and Behaviour, Radboud University Nijmegen, Nijmegen, The Netherlands; c Cognitive, Perceptual and Brain Sciences and Institute of Cognitive Neuroscience, University College London, London, UK

**Keywords:** Learning, Auditory systems, Functional MRI, Prefrontal cortex, Temporal cortex

## Abstract

Listeners show remarkable flexibility in processing variation in speech signal. One striking example is the ease with which they adapt to novel speech distortions such as listening to someone with a foreign accent. Behavioural studies suggest that significant improvements in comprehension occur rapidly — often within 10–20 sentences. In the present experiment, we investigate the neural changes underlying on-line adaptation to distorted speech using time-compressed speech. Listeners performed a sentence verification task on normal-speed and time-compressed sentences while their neural responses were recorded using fMRI. The results showed that rapid learning of the time-compressed speech occurred during presentation of the first block of 16 sentences and was associated with increased activation in left and right auditory association cortices and in left ventral premotor cortex. These findings suggest that the ability to adapt to a distorted speech signal may, in part, rely on mapping novel acoustic patterns onto existing articulatory motor plans, consistent with the idea that speech perception involves integrating multi-modal information including auditory and motoric cues.

## Introduction

When meeting someone with a heavy foreign or regional accent, listeners may find themselves struggling to understand them at first, but comprehension becomes easier within a few minutes. After interacting even longer, one may not even be aware of the speaker's accent anymore. This situation illustrates a remarkable faculty of the speech comprehension system: the ability to quickly adapt to the acoustic consequences of a wide variation in sources when perceiving speech. Listeners have been found to adapt to foreign-accented speech (Clarke and Garrett, 2004), noise-vocoded speech (Shannon et al., 1995), spectrally shifted speech (Rosen et al., 1999), synthetic speech (Greenspan et al., 1988), and time-compressed speech (Dupoux and Green, 1997) to name a few. This ability to adapt to distortions of the speech signal in general, has been studied extensively using time-compressed speech, which is a method for artificially shortening the duration of an audio signal without affecting the fundamental frequency of that signal (Golomb et al., 2007; Pallier et al., 1998; Sebastián-Gallés et al., 2000; Wingfield et al., 2003). Listeners are quickly able to adapt to sentences compressed up to 35% of their original duration, within 10–20 sentences (Dupoux and Green, 1997). Even though the distortion of the acoustic signal associated with time-compressing speech differs from, for instance, variations caused by speaking with a foreign accent, time-compressed speech has been used to study adaptation processes, as it is easy to create speech samples at a wide variety of compression rates. Furthermore, it allows for using the same speaker in time-compressed and uncompressed conditions, which is often not possible using foreign-accented speech.

Behaviourally, perceptual adaptation to distorted speech has often been described as an attention-weighing process in which listeners shift their attention from task-irrelevant to task-relevant cues (Goldstone, 1998; Golomb et al., 2007; Nosofsky, 1986). More specifically, it has been argued that learning of time-compressed speech is characterised by the recalibration of the boundaries between speech sounds to accommodate the faster speech rate (Golomb et al., 2007). In other words, the adaptation is believed to occur primarily at an auditory level using increased attentional resources.

Although previous studies have investigated the neural bases associated with comprehending time-compressed speech, none have investigated the adaptation process that occurs when listeners are first confronted with this unusual manipulation of the speech stream. For instance, Peelle et al. (2004) reported that processing time-compressed speech strongly recruits bilateral auditory cortices, among other regions. Similarly, Poldrack et al. (2001) found that activation in left superior temporal sulcus (STS) increased as sentence compression rate increased up to 30% of the original duration. Further compression, however, rendered the speech unintelligible and reduced activation in left STS. In contrast, right STS activation increased linearly, even at the highest levels of compression where speech was no longer intelligible. These results suggest two different processing mechanisms: a left hemisphere linguistic component responding to the content of the sentences and a right hemisphere acoustic component responding primarily to the complexity of the acoustic signal. Neither study, however, investigated the adaptation process — in fact, Peelle et al. familiarised listeners with the sound of time-compressed speech prior to taking part in the fMRI experiment specifically to avoid this confound. As a result, it is unclear which neural systems are responsible for this rapid perceptual adaptation.

In the present study, we aimed to address this question by monitoring the on-line adaptation process while participants performed a speeded sentence verification task on time-compressed sentences. The goal of the present study, therefore, was to better understand the neural mechanisms underlying the adaptation process itself, instead of the specific neural activation pattern for processing time-compressed speech, as was the case in previous studies.

In the field of speech comprehension research, there has been a longstanding debate about which mechanisms are required for speech processing. One theory holds that only auditory processes are required for effective speech perception (Diehl and Kluender, 1989; Stevens and Klatt, 1974). A competing theory claims there is an additional role for the motor (i.e. speech production) system and is derived from Liberman's Motor Theory of Speech Perception (Liberman et al., 1967; Liberman and Mattingly, 1985). In its original form, MTSP claimed: first, that speech tokens such as words, phonemes or phonetic features can only be recognized by mapping acoustic patterns onto articulatory (motor) plans and second, that speech processing involves a tight coupling between auditory and motor processes. The former claim is clearly incorrect (Diehl and Kluender, 1989; Galantucci et al., 2006; Toni et al., 2008). Recent studies, however, have found support for a tight coupling between perception and production systems (Liberman and Whalen, 2000) by showing the involvement of the speech motor system in speech perception tasks (D'Ausilio et al., 2009; Davis and Johnsrude, 2003; Fadiga et al., 2002; Okada and Hickok, 2006; Pulvermüller et al., 2006; Watkins et al., 2003). Meister et al. (2007) provided perhaps the clearest evidence by using transcranial magnetic stimulation to show that stimulation of left ventral premotor cortex (PMv) disrupted speech perception when syllables were embedded in noise without affecting a similar control task of detecting tones in noise. In short, there is renewed interest in the involvement of the motor (i.e. articulatory) system in speech perception, although not in the form of Liberman's original Motor Theory.

Crucially, the two accounts make different predictions about the neural mechanisms involved in the adaptation process. The former predicts that it is done purely acoustically by recognizing distorted signals as instances of abstract auditory prototypes such as phonemes or phonological word forms. Consequently, adaptation-related activation changes would be expected solely in auditory regions associated with speech perception (Guenther et al., 2004). The latter, however, predicts that the distorted acoustic signal is recognized at least in part by mapping it onto articulatory motor plans — a form of sensorimotor integration that implicitly simulates the motor patterns used to produce a comparable spoken sentence. In this case, adaptation-related activation would be expected in both auditory regions as well as in ventral premotor regions associated with speech production (Blank et al., 2002; Watkins et al., 2003, 2002).

## Materials and methods

### Participants

Twenty-two participants (13M, 9F) took part in the study although four (2M, 2F) were subsequently excluded due to: i) excessive head motion (> 10 mm), ii) an unexpected brain abnormality, iii) chance level performance in the scanner, and iv) an error acquiring the scanning data. The 18 remaining participants were right-handed, native speakers of British English (mean 26.7 years, median 22.5 years, range 18–60 years) without any history of neurological or psychiatric disease. The behavioural and neuroimaging data from the older participant (the one 60 year-old) did not differ qualitatively from the younger participants and therefore was included in all analyses. None had any form of oral or written language impairment or any previous experience with time-compressed speech. None of the participants reported any hearing difficulties, but were not audiometrically screened. In-scanner preliminary testing revealed that all participants could hear the stimuli clearly enough to perform the task (see fMRI data acquisition, below). All gave written informed consent and were paid for their participation. The study was approved by the NHS Berkshire Research Ethics Committee.

### Task

The task was a computerized version of the Speech and Capacity of Language Processing Test, or SCOLP (Baddeley et al., 1992; May et al., 2001). Participants listened to a simple sentence and decided whether it was true or false, indicating their response with a button press. In all cases, the validity of the sentence was obvious (e.g., “Bedroom slippers are made in factories” vs. “Nuns are made in factories”) with invalid sentences generated by changing participants and predicates from true sentences (see Adank et al. (2009) for additional task details). Accuracy and response times were recorded per trial and adaptation to time-compressed speech was operationalized as the increase in the speed of sentence verification times.

### Stimuli

The auditory stimuli were recordings of 200 SCOLP sentences, 100 true and 100 false, by a male Southern Standard British English speaker. The recordings were made in an anechoic room directly onto digital auditory tape (DAT), while the digital output from the DAT recorder was fed to the digital input of the sound card in the PC. Next, all sentences were saved into separate files with the beginning and ends trimmed at zero crossings as closely as possible to the onset/offset of the initial/final speech sounds and re-sampled at 22050 Hz. The time-compressed sentences were obtained using PSOLA (Moulines and Charpentier, 1990), as implemented in the Praat software package (Boersma and Weenink, 2003). Two versions of each recorded sentence were created: sentences resynthesized at 100% of their original duration (normal-speed sentences) and resynthesized sentences shortened to 45% of their original duration (time-compressed sentences). The normal sentences were resynthesized to ensure that any differences between the two types of sentences were due solely to the time compression and not the resynthesis process. The sentences consisted of 6.5 syllables on average (range 3–12 syllables, range 477–1221 ms) and the average speech rate of the normal-speed sentences was 4.1 syllables per second, and the average speech rate of the time-compressed sentences was 9.2 syllables per second. Finally, each sentence was peak-normalized at 99% of its maximum amplitude and scaled to 70 dB SPL using Praat. Stimulus presentation and response time measurement were performed using Presentation (Neurobehavioral Systems, Albany, CA).

### Design and procedure

**Fig. 1:**
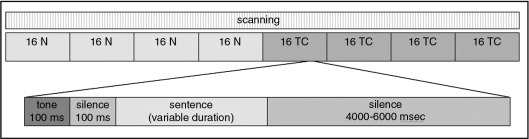
Presentation of blocks of normal-speed (N) sentences and time-compressed (TC) sentences (top) in the experiment, plus sequence of events in the presentation of one sentence (bottom).

The main experiment used an atypical block design in which all the normal-speed sentences (*n* = 64) occurred in the first half of the experiment and all the time-compressed sentences (*n* = 64) in the second half. This was necessary because pilot testing in the scanner demonstrated that alternating blocks of normal-speed and time-compressed sentences during scanning prevented behavioural adaptation — in fact, participants found both types of speech much more difficult. Consequently, the design shown in Fig. 1 was used to allow listeners to get used to the task and to the scanner noise during the presentation of the 64 normal-speed sentences and to allow them to efficiently tune into the time-compressed sentences in the second block. Such a design is similar to pharmacological fMRI studies where the time course of the pharmacological agent often makes it impossible to alternate between drug and non-drug conditions. Like those studies, we specifically looked for interactions between our experimental conditions and time to exclude non-specific effects of time such as scanner drift and physiological noise aliasing (Wise and Tracey, 2006).

A single trial began with a tone signal of 100 ms, followed by a pause of 100 ms, and then the auditory sentence (Fig. 1). The inter-trial interval varied randomly between 4000–6000 ms providing a jittered sampling of the evoked haemodynamic response function (Dale, 1999; Veltman et al., 2002). Although the stimuli were presented and analysed in an event-related design, trials occurred in short mini-blocks of four sentences followed by a silent baseline trial (duration randomly varied from 4000–6000 ms) to maximize statistical power. The entire duration of the run was 17 min.

Afterwards, a second (behavioural) test was run outside the scanner to determine whether adaptation was stable after the scanning session or whether it continued in the quieter environment. Participants were tested individually in a quiet room using headphones (Philips SBC HN110) immediately following the fMRI experiment. 64 new time-compressed sentences were presented. Presentation of all three sets of 64 sentences (normal-speed, time-compressed, and the time-compressed sentences in the post-task) was counter-balanced across subjects. Each set consisted of 32 true and 32 false sentences. The sentences were presented in a semi-randomised order per participant and true and false sentences were counter-balanced across experimental blocks.

### fMRI data acquisition

Scanning was performed at the Birkbeck-UCL Neuroimaging (BUCNI) Centre on a 1.5 T MR scanner (Siemens Avanto, Siemens Medical Systems, Erlangen, Germany). The experiment began by acquiring a high-resolution structural scan (3D Turbo-FLASH, TR = 12 s, TE = 5.6 ms, 1 × 1 × 1 mm resolution) used for anatomical localisation. Next, participants were familiarised with the task during a brief practice run using six normal-speed sentences not included in the rest of the experiment. They were instructed to respond through a button press with their right index finger when the sentence was true and with their right middle finger when the sentence was false. The sentences were presented over electro-static headphones (MRConFon, Magdeburg, Germany) during continuous scanner acquisition (GE-EPI, TR = 3 s, TE = 50 ms, 192 × 192 FOV, 64 × 64 matrix, 35 axial slices, yielding a notional 3 × 3 × 3 mm resolution) — in other words, over the noise of the scanner. The main experiment lasted just under 17 min and on average, 332 volumes (range: 330–336) were collected per participant. The presentation of the four blocks of normal sentences lasted on average 172 volumes (43 per block) for the normal-speed sentences, and 151 volumes (38 per block) for the time-compressed sentences. In other words, slightly less data were collected for the time-compressed sentence conditions because, by definition, the duration of the sentences was shorter. In theory, this may slightly reduce BOLD signal sensitivity for time-compressed relative to normal-speed sentences, but this was unavoidable given the nature of the experiment.

The choice of continuous, rather than sparse, sampling was based on a trade-off between the ability to reliably detect adaptation-related changes in blood oxygen level dependent (BOLD) signal and the length of the experiment. Continuous sampling results in both acoustic masking of the auditory sentences (Shah et al., 1999) and contamination of the BOLD signal response in auditory regions (Bandettini et al., 1998; Hall et al., 1999; Talavage et al., 1999). The former, however, was not a problem as a relatively quiet acquisition sequence (∼ 80 dB SPL) coupled with sound attenuating headphones (∼ 30 dB attenuation) ensured that the sentences were easily heard. Indeed, all participants confirmed their ability to hear and understand the sentences during this practice session. Contamination of the BOLD signal was potentially more problematic because scanner noise elevates BOLD responses in auditory areas (Gaab et al., 2006; Hall et al., 1999), and these effects need not be identical across regions (Tamer et al., in press; Zaehle et al., 2007). In the current experiment, however, we were specifically interested in reductions in BOLD signal that index adaptation-related changes. As a result, elevated BOLD responses *per se* were not problematic; only responses driven to saturation levels by the scanner noise would reduce sensitivity and previous studies have clearly shown that typical EPI sequences reduce, but do not eliminate, the dynamic range of the BOLD response (Gaab et al., 2006; Zaehle et al., 2007). Moreover, although some “silent” imaging protocols exist (Hall et al., 2009; Schwarzbauer et al., 2006) they are not yet widely available. fMRI systems without these protocols (such as our own) require silent periods between volume acquisitions lasting between 16 and 32 s to avoid scanner-noise contamination and ensure an adequate sampling of the evoked haemodynamic response function (HRF) (Eden et al., 1999; Edmister et al., 1999; Hall et al., 1999; Hickok et al., 1997; Tamer et al., in press). A sparse design would therefore result in our experiment lasting between 54 and 90 min, which was deemed likely to seriously reduce participant's performance due to fatigue. As a result, we chose to use a continuous sampling paradigm instead. One consequence of this choice was that the adaptation task was performed over the background noise of the scanner, and it became an empirical question whether embedding its noise would alter the typical behavioural profile of rapid adaptation.

### Analyses

Response times (RT) were measured from the end of each audio file, following May et al. (2001), and RTs beyond 3000 ms were trimmed without replacement (0.4%). Each set of sentences was divided into four blocks of 16 so that the time course of adaptation could be examined. A 16 sentence block-size was used because pilot testing revealed that learning was typically stable after 14–18 sentences and smaller windows reduce the accuracy of estimating induced BOLD signal responses (Murphy and Garavan, 2005). The mean RT of correct responses was used in the group analyses. Both accuracy and RTs were evaluated with a repeated-measures 2 × 4 ANOVA with Speech Type (normal-speed, time-compressed) and Block (1–4) as independent factors. Obviously, adaptation to the time-compressed sentences was only possible in the second half of the experiment. Consequently, our *a priori* hypothesis was that we would observe adaptation effects only for time-compressed and not normal-speed sentences.

The functional imaging data were analysed using FSL (www.fmrib.ox.ac.uk/fsl). After deleting the first three volumes of each run to allow for T1 equilibrium, the functional images were realigned to correct for small head movements (Jenkinson et al., 2002). The images were then smoothed with a 6 mm FWHM Gaussian filter and pre-whitened to remove temporal auto-correlation (Woolrich et al., 2001). The resulting images were entered into a subject-specific general linear model with eight conditions of interest: four blocks of normal-speed and four blocks of time-compressed sentences. Each sentence was convolved with a double gamma “canonical HRF” (Glover, 1999) to generate the regressors. The onset of this HRF function was aligned with the onset of every sound file and the duration of every sentence was included in the model. Temporal derivatives were also included to better fit small deviations in the expect time course. Both the data and the model were high-pass filtered at 1/200 s to remove low frequency signal drifts such as aliased cardiac or respiratory signals without affecting the more rapid, experimentally-induced frequencies such as those between mini-blocks and blocks of stimuli. Finally, each anatomical T1 scan was registered to the MNI-152 template using an affine transformation (Jenkinson and Smith, 2001), which was then applied to the first-level parameter and variance estimates. These were fed into a second-level mixed-effects analysis for inferring across the population (Beckmann et al., 2003; Woolrich et al., 2004).

Linear weighted contrasts were used to identify three effects of interest. First, the main effect of processing auditory sentences relative to scanner noise (when no sentence was presented) was computed to identify task-relevant brain regions using a contrast of [+ 1 + 1 + 1 + 1 + 1 + 1 + 1 + 1] where the first four conditions are the four blocks of normal-speed sentences and the last four of the blocks of time-compressed sentences. Significant activations were assessed with a cluster-based correction for multiple comparisons using a height threshold of *Z* > 3.5 and a corrected *p* < 0.05 cluster-extent (Friston et al., 1994). This corresponded to a minimum of 123 contiguous 2 × 2 × 2 mm voxels with *Z*-scores of 3.5 or greater. Next, we identified the regions within this system that were significantly more active for time-compressed relative to normal speech using the same statistical criteria and a contrast of [− 1/4 − 1/4 − 1/4 − 1/4 + 1/4 + 1/4 + 1/4 + 1/4]. Finally, the critical analysis aimed to identify areas within this system involved in adapting to time-compressed speech. To do this, we used a contrast that followed the behavioural profile of adaptation, namely greater activation for the first block of time-compressed sentences than the remaining three blocks [0 0 0 0 + 1 − ^1^/_3_ − ^1^/_3_ − ^1^/_3_]. It is worth noting, however, that this contrast may be confounded with linear time effects such as scanner drift or physiological noise that are not completely removed by high-pass filtering. Consequently, the contrast was inclusively masked (at *Z* > 3.5) by two other contrasts to ensure the effects were due to adaptation rather than scanner drift. The first was the main effect of sentence processing, to limit the results to areas specifically involved in the task [+ 1 + 1 + 1 + 1 + 1 + 1 + 1 + 1], while the second was the contrast between the first block of time-compressed sentences and the last block of normal-speed sentences [0 0 0 − 1 + 1 0 0 0]. Here we assumed that time-compressed sentences would significantly increase processing demands relative to normal sentences, consistent with the reaction time cost of switching to time-compressed sentences (see below). Importantly, this difference goes in the opposite direction to the time confound in the main contrast and helps to exclude activation unrelated to adapting to time-compressed speech. Significance was assessed using a small volume correction (Worsley et al., 1996) based on the smoothness and volume of the intersection of the inclusive masks. Within this reduced volume, a voxel-wise height threshold of *Z* > 3.0 corresponded to *p* < 0.05, one tailed. To illustrate the regional activation profiles, the mean parameter estimates per condition per participant were extracted from the activation cluster. Note that post-hoc t-tests are Bonferroni corrected to adjust for multiple comparisons except where correcting was less conservative (as stated in the text) and significance was assessed at *p* < 0.05.

## Results

### Behaviour

**Fig. 2:**
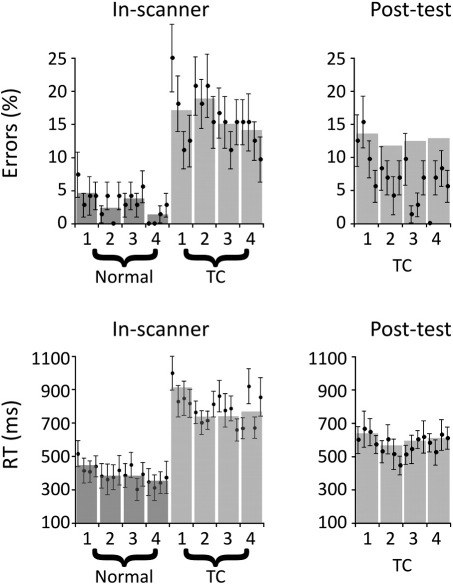
Behavioural results in the scanner (In-scanner) and outside the scanner in the post-test (Post-test) for the average error rates (top) and the average response times (bottom). Averages for the error rates and response times are represented per mini-block of 4 sentences (black error bars, 4 per block) and averaged across the blocks (1–4) of 16 sentences (underlying grey bars). The averages for blocks 1–4 (16 sentences per block) were used for analysing both the behavioural and functional imaging data. Filled circles represent a mean over subjects of a four-sentence mini-block and error bars represent standard error of the mean. Dark bars refer to normal-speed sentences and lighter bars indicate time-compressed (TC) sentences.

Fig. 2 shows the results for the error rates and the response times recorded inside and outside the scanner. In order to illustrate the time course of adaptation, data are shown averaged over each mini-block of four trials by a filled-in circle and error bars indicating the standard error of the mean. Data from four consecutive mini-blocks was then averaged and displayed as a bar plot. These bars corresponded to the blocks of 16 sentences used in the analyses. The data from the mini-blocks were included to provide a more fine-grained temporal resolution of the adaptation process than provided by the larger blocks of 16 sentences and are shown for illustration purposes only; all statistical analyses were performed on the averages across the larger blocks of 16 sentences only. What should be clear from the figure is that despite the noisy environment of the scanner, participants were able to perform the task well. Error rates for normal-speed sentences were only 3% while time-compressed sentences were more difficult, with an average error rate of 16%. This main effect of Speech Type was significant (*F*(1,17) = 196.8, *p* < 0.0001, partial *η*^2^ = 0.92), but the main effect of Block (*F*(3,51) = 1.7, *p* = 0.172, partial *η*^2^ = 0.09) and the interaction (*F*(3,51) = 1.1, *p* = 0.378, partial *η*^2^ = 0.06) were not. In order to determine whether there were any adaptation effects that may have been hidden by the omnibus ANOVA, the normal-speed and time-compressed sentences were re-analysed separately. Normal-speed sentences showed an effect of Block (*F*(3,51) = 5.6, *p* = 0.03, partial *η*^2^ = 0.25), indicating that participants got better at the task over time. Interestingly, there was no equivalent effect for time-compressed sentences (*F*(3,51) = 1.1, *p* = 0.306, partial *η*^2^ = 0.06), suggesting that by the time these sentences began, the participants were essentially acclimated to the task and environment. This is consistent with the fact that errors in the post-scan behavioural test did not significantly differ across blocks (*F*(3,51) = 2.1, *p* = 0.115, partial *η*^2^ = 0.11). In other words, the error rates suggest that participants successfully acclimated to the task within the noisy environment of the scanner before the time-compressed sentences were introduced.

The analysis of RTs revealed a main effect of Speech Type (*F*(1,17) = 80.8, *p* < 0.0001, partial *η*^2^ = 0.83), indicating that responses to time-compressed sentences were significantly slower than normal-speed sentences (790 vs. 386 ms). There was also a main effect of Block (*F*(3,51) = 5.0, *p* = 0.004, partial *η*^2^ = 0.23) that was largely driven by the slowed responses to the first block of time-compressed sentences. When the two types of speech types were analysed separately, normal-speed sentences showed no effect of Block (*F*(3,51) = 1.58, *p* = 0.226, partial *η*^2^ = 0.09), indicating that listeners did not vary significantly in their response times across the four blocks. On the other hand, the analysis of the time-compressed sentences did reveal a significant effect of Block (*F*(3,17) = 5.0, *p* = 0.004, partial *η*^2^ = 0.23). A series of planned *t*-tests showed that responses to the first block of time-compressed sentences were significantly longer than those to subsequent blocks (all paired *t*-tests, *p* < 0.05). In other words, within the first 16 trials, participants had adapted to the atypical speech signal as evidenced by the fact that RTs in the subsequent blocks were on average 150 ms faster than the first block of time-compressed sentences. This was further confirmed when the RTs in the post-scan behavioural test did not show a significant effect of Block (*F*(3,17) = 2.34, *p* = 0.085, partial *η*^2^ = 0.13), confirming that adaptation asymptoted during the 64 trials that occurred within the scanner.

### Neuroimaging

To begin, we compared blood oxygen level dependent (BOLD) signal across the eight auditory sentence task conditions to fixation in order to identify the system of regions involved in the task. Like previous studies (Binder et al., 2000; Crinion et al., 2003; Giraud et al., 2004; Price et al., 2005; Scott et al., 2000), we found robust activation in primary auditory and auditory association cortices bilaterally, as well as in the deep frontal operculum bilaterally, left prefrontal and premotor cortices, and pre-SMA extending ventrally into the cingulate sulcus (see Table 1 for the complete list). The activation in the operculum bilaterally corresponds with earlier findings reported on processing degraded/noisy speech input (Benson et al., 2001; Wong et al., 2002; Davis and Johnsrude, 2003; Giraud et al., 2004; Meyer et al., 2004).

**Fig. 3:**
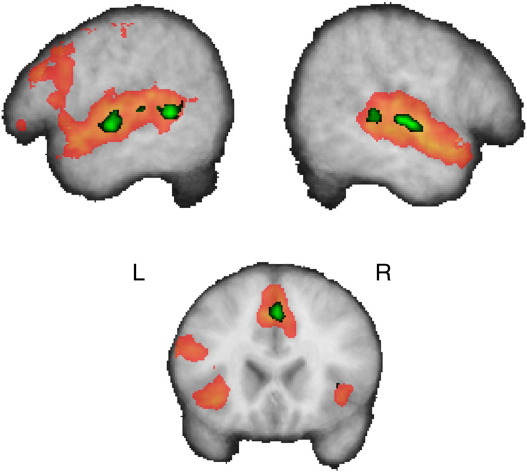
Activation for time-compressed relative to normal sentences (green) is superimposed on activation for all sentences relative to the fixation baseline (red) and shown on the mean structural image from the 18 participants.

Next, we identified the regions within this system that were more active for time-compressed relative to normal speech (Table 2A and Fig. 3). This revealed separate posterior and anterior temporal lobe foci bilaterally. One was located in the superior temporal sulcus (STS) posterior to Heschl's gyrus while the other was located in the lateral portion of Heschl's gyrus as it joins the anterior superior temporal gyrus (STG). In other words, time-compressed sentences produced greater levels of activation in STS and STG compared to normal-speed sentences, consistent with previous reports that activation in these regions increased with the level of speech compression (Peelle et al., 2004; Poldrack et al., 2001). In addition, we observed activation in pre-SMA (0, + 12, + 60, *Z* = 4.0).

**Fig. 4:**
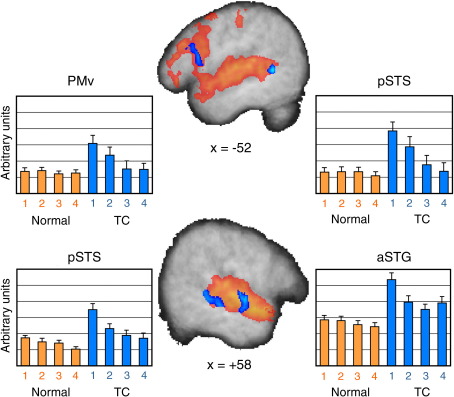
Adaptation-related activation patterns in the left (top row) and right (bottom row) hemispheres are shown in blue superimposed on all sentences relative to baseline (red). For each of the four regions, bar plots indicate changes in BOLD signal relative to baseline for the four blocks of normal sentences (orange) and the four blocks of time-compressed sentences (blue).

Of primary interest, however, were the neural changes associated with adapting to time-compressed speech (Table 2B). Four regions showed adaptation-related activation profiles, illustrated in Fig. 4. In the left hemisphere, one cluster was located within a region of the ventral bank of posterior STS. Within this area, there was a significant main effect of Stimulus Type (*F*(1,17) = 15.5, *p* = 0.001, partial *η*^2^ = 0.48) indicating greater activation for compressed relative to normal speech, a main effect of Block (*F*(3,51) = 5.0, *p* = 0.004, partial *η*^2^ = 0.23) and a significant interaction (*F*(3,51) = 4.3, *p* = 0.008, partial *η*^2^ = 0.20). When the two types of speech were analysed separately, normal-speed sentences showed no effect of Block (*F*(3,51) = 0.68, *p* = 0.568, partial *η*^2^ = 0.04), indicating that activation was greater than baseline and stable over all four blocks, mirroring the response time findings. The time-compressed sentences, however, did show an effect of Block (*F*(3,51) = 5.1, *p* = 0.004, partial *η*^2^ = 0.23) which was driven by a significant increase in the magnitude of the activation during the first block of time-compressed sentences (*t*(17) = 4.6, *p* < 0.001). Activation levels in the first block of time-compressed sentences more than tripled, signifying a considerable increase in processing demands. By the third block of time-compressed sentences, however, activation had returned to normal sentence processing levels (*t*(17) = 1.0, *p* = 0.319 uncorrected). A second left hemisphere cluster located on the crest of the pre-central gyrus, a region of ventral premotor cortex (PMv), showed essentially the same pattern. Again there were significant main effects of Stimulus Type (*F*(1,17) = 7.4, *p* = 0.014, partial *η*^2^ = 0.31) and Block (*F*(3,51) = 3.9, *p* = 0.014, partial *η*^2^ = 0.19), as well as a significant interaction (*F*(3,51) = 3.7, *p* = 0.016, partial *η*^2^ = 0.18). Normal sentences showed no effect of Block (*F*(3,51) = 0.38, *p* = 0.762, partial *η*^2^ = 0.02), whereas time-compressed sentences did (*F*(3,51) = 4.5, *p* = 0.007, partial *η*^2^ = 0.21). As in the pSTS region, activation levels increased significantly for the first block of time-compressed sentences (*t*(17) = 4.0, *p* = 0.004) and then returned to normal levels by the third block (*t*(17) = 0.4, *p* = 0.679 uncorrected). In short, the activation profile in both left pSTS and left PMv closely matched the response time data.

Adaptation-related changes in the two right hemisphere clusters, on the other hand, showed a slightly different pattern. The first region was located on the anterior crest of STG and extended into the dorsal bank of STS while the other was located more posterior in the ventral bank of STS. In both regions, activation was significantly greater than baseline for normal sentences but was not stable over the four blocks of normal sentences — instead it monotonically decreased. This was confirmed by a significant main effect of Block (both *F*(3,51) ≥ 35.0, *p* < 0.0001, partial *η*^2^ ≥ 0.67) which was also present when normal sentences were analysed separately (*F*(3,51) ≥ 2.8, *p* ≤ 0.050, partial *η*^2^ ≥ 0.14). In both regions, the first block of time-compressed sentences significantly increased activation levels (anterior: *t*(17) = 7.0, *p* < 0.001; posterior: *t*(17) = 5.7, *p* < 0.001), but only in the more posterior cluster did these return to normal levels (for blocks 3 and 4: *t*(17) = 1.8, 1.0, *p* = 0.092 and 0.352 uncorrected). In the more anterior region, activation remained significantly greater for all blocks of time-compressed relative to normal sentences (for blocks 2–4: *t*(17) = 4.6, 3.5, 3.8, all *p* ≤ 0.012 corrected). In sum, the activation in the left and right-lateralised regions increased strongly for the time-compressed sentences before showing a sharp decline after the first blocks of time-compressed speech. However, the left and right regions differed in their activation pattern for the normal sentences with activation in the left-lateralised regions remaining constant, while activation in the right-lateralised regions declined. This was confirmed by a significant Hemisphere × Block interaction (*F*(3,54) = 2.9, *p* = 0.045) for normal-speed sentences that used mean BOLD signal from the two left and two right hemisphere areas showing adaptation effects. It is worth noting that the theoretical question of reduced statistical sensitivity for time-compressed sentences appears not to be a major concern in practice, given the large effect sizes and small error variances seen in Fig. 4. In other words, the sensitivity was sufficient to detect significant BOLD signal effects for both time-compressed sentences as well as the effects of adaptation.

A final set of analyses investigated whether any of the results were related to the presence of semantic violations (by virtue of including true and false sentences together). This analysis modelled true and false sentences separately to avoid the potential confound associated with semantic violations. Although the effect sizes were smaller due to the lower number of cases, the results showed a pattern identical to the analysis with the true and false sentences combined. In other words, the activations associated with adapting to time-compressed speech cannot be attributed to semantic violations present in the false sentences.

## Discussion

The current results confirm and extend previous behavioural studies that demonstrate rapid on-line adaptation to atypical speech signals. After hearing just 16 sentences, participants' comprehension was both accurate and much faster than their initial responses to time-compressed speech, despite the concurrent, on-going noise of the MRI scanner. Moreover, the final behavioural test outside of the scanner demonstrated that the adaptation process completed within the first 64 trials as no additional learning took place after scanning. Instead, there was a trend towards slightly faster RTs which did not reach significance but may point to a re-tuning process that occurs after adaptation had been completed (Dupoux and Green, 1997), perhaps due to different acoustic environments (i.e. with or without scanner noise).

Adaptation-related changes in neural activation were observed in four separate areas: two in the right hemisphere and two in the left. In the right, the regions were both auditory association areas located in anterior and posterior portions of the STS, respectively. Both showed significant adaptation to normal sentences, with activation decreasing over the first four blocks. When time-compressed sentences were introduced, activation increased dramatically at first and then decreased after the first block, although it did not return to normal levels. This pattern of responses suggests that adaptation may have occurred at an acoustic, rather than linguistic, level for three reasons. First, the initial BOLD signal adaptation coupled with increasing accuracy rates for normal sentences appears to reflect a gradual acclimation to hearing sentences in a noisy environment. This may reflect adaptation occurring at an acoustic, rather than linguistic, level due to the energetic masking of the scanner noise (Aydelott and Bates, 2004). Second, the fact that the BOLD response did not return to levels associated with normal-speed sentences suggests that activation in these areas may be driven primarily by the condensed acoustic signal rather than by its content. This interpretation is consistent with the findings of Poldrack et al. (2001), who demonstrated a linear increase in activation within right STS with increasing time compression, even when the compression level rendered the auditory sentence unintelligible. Finally, a recent study reported stronger interactions between scanner noise and acoustic processing in right hemisphere auditory areas (Schmidt et al., 2008), suggesting greater sensitivity to acoustic over linguistic processing in right auditory cortex.

In contrast, adaptation-related changes in the left hemisphere may be more directly related to comprehending speech. In both pSTS and PMv, activation was stable over the first four blocks of normal sentences before increasing by 2–3 fold for the first block of time-compressed sentences. Activation then decreased to the levels seen for normal sentences. This pattern more closely matched the behaviour and subjective experience of the participants who reported no difficulty with the normal-speed sentences and no difficulty for the time-compressed sentences “once they got used to them.” Again, this result is consistent with those from Poldrack et al. (2001) who reported a convex response profile for activation in left STS. As time compression increased from 60% to 30% of the original sentence duration, BOLD signal increased. At 15% of a sentence's original duration it was no longer comprehensible and left STS activation reduced to baseline levels. Together with the current results, both studies suggest that adaptation in the left hemisphere regions appears to be at a linguistic level. Moreover, both studies highlight an apparent difference between right and left STS in responding to time-compressed speech: right STS appears to be driven more by the complexity of the acoustic signal while left STS responds more strongly to its linguistic content. This difference is consistent with the theoretical framework of Hickok and Poeppel (2007) in which two distinct anatomical streams are involved in processing speech signals. A ventral stream runs along the STS and is primarily concerned with the content of the speech signal while the dorsal stream links posterior auditory cortex to anterior motor regions involved in articulation. Critically, the latter is strongly left lateralised, includes both pSTS and PMv, and provides an anatomical substrate for mapping acoustic speech signals onto frontal lobe articulatory networks. With respect to sensorimotor interactions, it is proposed that sensorimotor integration is subserved by the dorsal stream.

The anatomy of regions involved in adaptation to time-compressed sentences helps to shed light on the nature of the adaptation mechanism. Specifically, pSTS is a region of auditory association cortex involved in speech perception (Binder et al., 1996; Scott et al., 2000; Scott and Wise, 2004; Wise et al., 2001), as well as perceiving other complex, non-linguistic sounds (Dick et al., 2007; Leech et al., 2009; Price et al., 2005). In contrast, the pre-central gyrus is part of the premotor cortex, which is involved in the selection and execution of complex motor sequences (Hoshi and Tanji, 2007; Rushworth et al., 2003). PMv, in particular, is closely linked with articulatory motor patterns due to its strong, reciprocal connectivity to the ventral areas of primary motor cortex, which enervate the face, larynx, and tongue (Barbas and Pandya, 1987; Brown et al., 2007). Speech production tasks robustly activate this region (Blank et al., 2002; Pulvermüller et al., 2006; Rauschecker et al., 2008, in press; Watkins et al., 2003). The fact that both sensory and motor areas demonstrate adaptation-related activation profiles, suggests that adapting to atypical speech involves changing sensitivity not only to auditory, but also to motoric cues. One possibility is that the novel acoustic patterns of compressed speech are mapped onto articulatory motor plans as an implicit form of motor simulation. This may aid in recognizing the speech tokens, particularly in challenging listening situations. Indeed, situations such as when the speech signal is either impoverished (Pulvermüller et al., 2006; Wilson et al., 2004), masked (Davis and Johnsrude, 2003; Meister et al., 2007) or ambiguous (Callan et al., 2004; Skipper et al., 2006) may preferentially recruit speech production regions to aid speech comprehension.

Adapting to time-compressed speech has often been classified as an attention-weighing process in which listeners learn to shift their attention from task-irrelevant to task-relevant auditory cues (Goldstone, 1998; Golomb et al., 2007; Nosofsky, 1986). Our results indicate that this specific form of perceptual learning may be supported by sensorimotor integration between auditory and speech production areas. For instance, the process of adjusting to distorted acoustic cues may place greater demands on verbal working memory, which engages this left sensorimotor circuit (Paulesu et al., 1993; Romero et al., 2006). This account is certainly consistent with the current findings showing increased PMv activation to the initial time-compressed sentences and raises the possibility that adapting to the compressed speech signal depends at least in partly on implicit articulatory simulation to recognize speech tokens in the atypical auditory input.

Finally, it is worth noting that the current findings are consistent with some, but not all, aspects of Liberman's MTSP (Liberman et al., 1967; Liberman and Mattingly, 1985). Like previous studies, our data demonstrate a clear role of auditory cortex in speech comprehension. According to the original MTSP, this auditory component would be a highly specialised speech-specific module separate from the rest of the auditory system and dedicated to conveying speech to the motor system where it would be identified as a series of articulatory gestures (Liberman and Mattingly 1985). In contrast, our data identifies a particular region of posterior STS that has also been shown to be involved in processing complex non-speech sounds (e.g., Leech et al., 2009) and thus runs counter to a core theoretical claim of MTSP. Our finding of significant activation in PMv when adapting to time-compressed speech, on the other hand, tends to support the notion that speech production regions may be preferentially recruited to aid speech comprehension in challenging listening situations such as a distorted, degraded or masked speech signal (Callan et al., 2004; Skipper et al., 2006). For example, Meister et al. (2007) showed that transcranial magnetic stimulation (TMS) to left PMv disrupted speech perception when syllables were embedded in noise without affecting a similar control task of detecting tones in noise. Additional studies are, however, required to establish whether these regions are *essential* for the adaptation process.

In summary, although ideal listening conditions facilitate speech perception, they rarely occur in real life. Normal environments are noisy with poor acoustics that degrade an incoming speech signal. As a result, the human speech recognition system seems to have evolved an opportunistic decoding approach that takes advantage of whatever information is available to assist in comprehension. Primarily this relies on auditory information, but other systems including vision (McGurk and MacDonald, 1976), somatosensation (Ito et al., 2009) and the motor system (D'Ausilio et al., 2009) may provide important additional cues as well.

## Figures and Tables

**Table 1:** Activation for auditory sentences (normal-speed and time-compressed) relative to the fixation baseline.

Region	Hemisphere	Peak coordinate			*Z*-score
Temporal lobe					
STG/STS	L	− 58	− 16	− 6	6.6
STG/STS	R	+ 62	− 12	− 8	7.0
Frontal					
Frontal operculum	L	− 32	+ 22	− 6	5.5
Frontal operculum	R	+ 40	+ 22	− 8	4.7
Pars opercularis	L	− 48	+ 12	+ 18	5.4
Pars orbitalis	L	− 52	+ 32	− 8	4.6
Pre-SMA	B	± 4	+ 2	+ 48	5.4
Cingulate sulcus	B	0	+ 22	+ 40	5.9
Parietal					
Anterior SMG	L	− 42	− 22	+ 50	4.6
Subcortical					
Thalamus	L	− 8	− 16	0	4.9
Medial geniculate body	B^a^	− 10	− 28	− 10	4.2

Coordinates are in MNI standard space.

a This activation presented bilaterally but was only significant in the left hemisphere where its extent blurs into the other thalamic activation.

**Table 2:** Activation associated with time-compressed sentences.

Region	Hemisphere	Peak coordinate			*Z*-score
A. Time-compressed relative to normal sentences					
Anterior STG/STS	L	− 60	− 14	0	4.7
Posterior STG/STS	L	− 58	− 46	+ 4	4.7
Anterior STG/STS	R	+ 64	− 14	0	4.7
Posterior STG/STS	R	+ 56	− 32	+ 4	4.0
Pre-SMA	B	0	+ 12	+ 60	4.3
Cingulate sulcus	B	0	+ 22	+ 44	4.3
Frontal operculum^a^	L	− 36	+ 24	− 4	3.4
Frontal operculum^a^	R	+ 36	+ 25	+ 2	4.1
B. Adaptation-related changes					
Posterior STS	L	− 54	− 52	2	3.6
Ventral premotor	L	− 50	+ 14	+ 12	3.1
Anterior STG	R	+ 58	− 8	− 4	3.3
Posterior STS	R	+ 64	− 40	0	3.5

Coordinates are in MNI standard space.

a Although there was activation in the frontal operculum, it was not extensive enough to reach significance using the cluster test and here is reported only for completeness.
